# The risk factors for burnout among nurses: An investigation study

**DOI:** 10.1097/MD.0000000000039320

**Published:** 2024-08-23

**Authors:** Kunyu Wang, Xia Wang, Ying Han, Chunfen Ye, Lifen Pan, Changcai Zhu

**Affiliations:** a Nursing Department, Wuhan University of Science and Technology Affiliated Tianyou Hospital, Wuhan, China; b Hemodialysis Center, Taikang Tongji (Wuhan) Hospital, Wuhan, China; c Nursing Department, Wuhan Hankou Hospital, Wuhan, China; d Neurosurgery, Wuhan University of Science and Technology Affiliated Tianyou Hospital, Wuhan, China; e Medical Imaging Department, Wuhan University of Science and Technology Affiliated Tianyou Hospital, Wuhan, China; f Department of Public Health and Preventive Medicine, School of Medicine, Wuhan University of Science and Technology, Wuhan, China.

**Keywords:** burnout, COVID-19, MBI-GS, nurses, risk factors

## Abstract

The objective of this investigation study is to examine the levels of burnout and work engagement among nurses working on the front lines of the COVID-19 pandemic. Additionally, we aim to analyze the risk factors associated with nurse burnout. This investigation study included a sample of 1764 registered nurses from 6 tertiary comprehensive hospitals in Wuhan. A total of 1800 questionnaires were distributed via email between January 2021 and July 2021, and 1764 completed questionnaires were returned. Nurses who had been on the front lines of the COVID-19 fight for more than six months were included in the study. The Maslach Burnout Inventory-General Survey (MBI-GS) scale was utilized to assess burnout levels among all nurses. Work engagement was measured using the Utrecht Work Engagement Scale (UWES). The general health of the nurses was evaluated using the General Health Questionnaire-12 (GHQ-12) score. Demographic and clinical data, including age, sex, hospital, department, education, years of experience, daily overtime, weekly rest time, number of night shifts per month, smoking or drinking habits, marital status, etc, were also collected. Statistical analyses were performed using SPSS 25.0. The GHQ-12 scores in the FC group were significantly higher than those in the non-frontline COVID-19 (NFC) group. Compared to the average value of the 2 groups, we found that the dimension 2 average value of UWES in NFC group (3.52 ± 1.07) were remarkably higher than that in FC group (3.40 ± 1.08, *P* < .05). Furthermore, the dimension 1 (emotional exhaustion) average value and dimension 2 (depersonalization) average value of MBI-GS in NFC group were remarkably declined than that in FC group. Spearman rank correlation showed that negative correlation among the average value of each dimension and the overall average values between MBI-GS and UWES. Logistic regression analysis showed that daily Overtime, fight against COVID-19, GHQ-12 score and dimension 2 of UWES were the risk factors for nurse burnout. In summary, this study showed that the dimension 1 (emotional exhaustion) average value and dimension 2 (depersonalization) average value of MBI-GS in NFC group were remarkably declined than that in FC group. This study may provide some basis for addressing nurse burnout.

## 1. Introduction

American psychoanalyst Freudenberger first proposed the concept of “work burnout” in 1974, referring to the long-term fatigue and physical and mental exhaustion resulting from work pressure.^[1]^ His phenomenon has been recognized as a chronic stress-related problem by the World Health Organization, particularly prevalent in the service industry and healthcare sector.^[2]^ Work burnout among nurses has become a significant concern as it not only affects their well-being but also impacts the quality of patient care.^[3–5]^ The factors that influence work burnout are numerous and complex and involve many external factors such as social and cultural backgrounds.^[6]^ They are also subject to current medical models, hospital policies, and other difficult-to-control factors.^[7]^ Perhaps shifting the focus to controllable internal factors of work burnout would be more helpful in addressing these issues.^[8]^

Nurses often face high levels of stress, which can lead to burnout.^[9]^ In recent years, nurse burnout has become a significant concern because it not only affects the nurses themselves, but also the quality of care they provide to patients.^[10]^ Previous studies have shown that factors influencing nurse burnout include long work hours,^[11]^ lack of control over the work environment,^[12]^ inadequate staffing,^[13]^ low job satisfaction,^[14]^ and poor relationships with colleagues and supervisors.^[15,16]^ Additionally, nurses often face emotional and physical pressures, which can contribute to burnout.^[17]^ In recent years, the outbreak of the COVID-19 pandemic in China has had a significant impact on healthcare workers, including nurses. They have been at the forefront of caring for COVID-19 patients, which has increased their risk of job burnout due to fear of infection, long working hours, and high levels of stress.^[18]^ Galanis et al analyzed the risk factors for nurse burnout during the COVID-19 pandemic and showed that inadequate preparedness for the outbreak, increased perception of the threat of COVID-19, long working hours in isolation wards, and working in high-risk environments can lead to nurse burnout.^[19]^ However, further research is needed to confirm the impact of frontline participation in the fight against COVID-19 on nurse burnout.

In this investigation study, we intend to gain insight into the burnout and work engagement among nurses who are working on the front lines of the COVID-19 pandemic and analyze the risk factors associated with nurse burnout. This study may provide some basis for addressing nurse burnout.

## 2. Methods

### 2.1. Subjects

This multicenter investigation study selected 1764 registered nurses from 6 tertiary comprehensive hospitals in Wuhan, including Tianyou Hospital affiliated to Wuhan University of Science and Technology, Hankou Hospital of Wuhan City, Third People’s Hospital of Hubei Province, Hubei Provincial Hospital of Integrated Traditional Chinese and Western Medicine, Liyuan Hospital affiliated to Tongji Medical College, and Wuhan Taikang Tongji Hospital, as the research subjects. A total of 1800 questionnaires were distributed by email from January 2021 to July 2021, and returned 1764 questionnaires, 52 invalid questionnaires, and 1712 valid questionnaires. All study subjects were divided into 2 groups based on whether they were frontline nurses in the fight against COVID-19: the frontline COVID-19 (FC) group and the non-frontline COVID-19 (NFC) group. Nurses in the fight against COVID-19 was defined as more than 6 months on the front lines of COVID-19. The inclusion criteria were as follows: hold a Nurse Practicing Certificate issued by the People’s Republic of China; work experience ≧ 1 year; regular employees of the hospital. The exclusion criteria include: internships, traineeships, and returning nurses; received psychological or medication treatment within 3 months. The study was approved by the Ethics Committee of Wuhan University of Science and Technology Affiliated Tianyou Hospital (No. TYHP-20220056). All participants agreed to participate in this study and signed an informed consent form.

For calculation of sample size, the formula of (*Z*_1−α/2_ * σ)^2^/δ^2^ was used. The mean Maslach Burnout Inventory-General Survey (MBI-GS) score of nurses were about 35 ± 12 according to the clinical experience and allowable error was 0.2. Thus α = 0.05, *Z*_1−α/2_ = 1.96, σ = 12, δ = 2. And the minimal sample size was 1411.

### 2.2. Data collection

Demographic and clinical statistics including age, sex, hospital, department, education, working age, daily overtime, weekly rest time, night shifts per month, smoking or drinking condition, marital status, etc were collected. We used the MBI-GS scale to assess burnout in all nurses.^[20]^ The scale contains 15 entries in 3 dimensions: 1 to 5 entries for emotional exhaustion (dimension 1), 6 to 9 entries for depersonalization (dimension 2), and 10 to 15 entries for personal accomplishment (dimension 3). The higher the score, the more severe the burnout. In addition, we assessed the work engagement of the nurses using the Utrecht Work Engagement Scale (UWES),^[21]^ which consists of 17 entries in 3 dimensions (dimension 1 vitality, dimension 2 dedication, dimension 3 focus), with higher scores associated with higher levels of work engagement. Finally, we rated the general health of nurses using the General Health Questionnaire-12 (GHQ-12).^[22]^

### 2.3. Statistical analysis

The SPSS 25.0 statistical software (IBM, Armonk) was used to establish the database. The normal distribution of data was confirmed by Kolmogorov–Smirnov analysis. Normal distribution data were expressed by mean ± SD while non-normal distribution data median (range). Mann–Whitney test or Student *t* test was used for comparison between 2 groups. Multivariate analysis of variance (MANOVA) was used to analyze the sub-scores based on different independent variables. Chi square test was used for rates. Spearman rank correlation was used for correlation analysis. Logistic regression was performed for risk factors of nurse burnout. *P* < .05 for the difference was considered statistically significant.

## 3. Results

### 3.1. Basic characteristics of all subjects

In this multicenter investigation study, a total of 1800 questionnaires were distributed by email, among returned 1764 questionnaires, 52 invalid questionnaires, and 1712 valid questionnaires, with an effective rate of 97.05%. The general demographic information of all nurses who participated in the investigation study was shown in Table S1, Supplemental Digital Content, http://links.lww.com/MD/N392. All subjects were divided into FC group (n = 1186) and NFC group (n = 526) based on whether they were frontline nurses in the fight against COVID-19. The basic characteristics of the 2 groups were shown in Table 1, the age of nurses in FC group was significantly older than that in the NFC group (*P* < .05). Compared with the NFC group, the working age of nurses in FC group were remarkably longer (*P* < .05). In addition, the proportion of nurses in the FC group who were married and the GHQ-12 score were significantly higher than those in the NFC group (*P* < .05). No other significant difference was found between 2 groups.

**Table 1:** Basic characteristics of all subjects.

Variables	NFC group, n = 526	FC group, n = 1186	*P*
Hospital			
Tianyou Hospital affiliated to Wuhan University of Science and Technology	76 (14.45)	251 (21.16)	.264
Wuhan Taikang Tongji Hospital	111 (21.10)	120 (10.12)	.052
Hankou Hospital of Wuhan	58 (11.03)	195 (16.44)	.408
Liyuan Hospital affiliated to Tongji Medical College	72 (13.69)	179 (15.10)	.843
Hubei Provincial Hospital of Integrated Traditional Chinese and Western Medicine	140 (26.62)	211 (17.79)	.175
Third People’s Hospital of Hubei Province	69 (13.12)	230 (19.39)	.335
Age	27.5 (20–55)	30 (21–59)	<.001
Sex, female n (%)	493 (93.72)	1164 (98.15)	.279
Department			
Internal medicine, n (%)	146 (27.76)	374 (31.53)	.644
Surgical, n (%)	114 (21.67)	265 (22.34)	1.000
Pediatrics, n (%)	17 (3.23)	41 (3.46)	1.000
Obstetrics, n (%)	36 (6.84)	57 (4.81)	.076
Other, n (%)	213 (40.49)	449 (37.86)	.885
Education			
Junior college or below, n (%)	163 (30.99)	247 (20.83)	.146
Bachelor or above, n (%)	363 (69.01)	939 (79.17)	.146
Working age	2 (1–5)	3 (1–5)	<.001
Daily overtime (h)	2 (1–4)	2 (1–4)	.092
Weekly rest time (d)	2 (1–3)	2 (1–3)	.197
Night shifts per month			
≤5, n (%)	287 (54.56)	668 (56.32)	1.000
≥6, n (%)	239 (45.44)	518 (43.68)	
Smoking or drinking, n (%)	12 (2.28)	26 (2.19)	1.000
Marital status			
Married, n (%)	287 (54.56)	427 (36.00)	.010
Unmarried, n (%)	230 (43.73)	739 (62.31)	.016
Divorced/widowed, n (%)	9 (1.71)	20 (1.69)	1.000
GHQ-12	13 (8–21)	17 (9–39)	.006

FC = frontline COVID-19, GHQ-12 = General Health Questionnaire-12, NFC = non-frontline COVID-19.

### 3.2. Comparisons of UWES scores between 2 groups

Further, we used UWES to assess the work engagement of the nurses. UWES consists of 3 dimensions with 17 entries. After measuring the score of each dimension, we divide it by the total number of entries in that dimension to obtain the average value. Compared the average value of the two groups, we found that the dimension 2 average value of UWES in NFC group (3.52 ± 1.07) were remarkably higher than that in FC group (3.40 ± 1.08, Table 2, *P* < .05).

**Table 2:** Compared the UWES average value between two groups.

UWES	NFC group, n = 526	FC group, n = 1186	*P*
Dimension 1	3.23 ± 1.00	3.18 ± 1.00	.394
Dimension 2	3.52 ± 1.07	3.40 ± 1.08	.037
Dimension 3	3.33 ± 0.99	3.27 ± 1.05	.244
UWES average	3.35 ± 0.96	3.28 ± 0.98	.156

FC = frontline COVID-19, NFC = non-frontline COVID-19, UWES = Utrecht Work Engagement Scale.

### 3.3. Comparisons of MBI-GS scores between 2 groups

We subsequently used the MBI-GS scale to evaluate the level of burnout in both groups of nurses. As shown in Table 3, compared the average value of the 2 groups, the dimension 1 average value and dimension 2 average value of MBI-GS in NFC group were remarkably declined than that in FC group (*P* < .05). Furthermore, Spearman rank correlation was used for correlation analysis. The results showed that negatively correlation among the average value of each dimension and the overall average values between MBI-GS and UWES (Table 4, *P* < .001).

**Table 3:** Compared the MBI-GS average value between two groups.

MBI-GS	NFC group, n = 526	FC group, n = 1186	*P*
Dimension 1	2.19 ± 1.10	2.32 ± 1.10	.021
Dimension 2	1.66 ± 1.13	1.80 ± 1.12	.014
Dimension 3	2.84 ± 1.12	2.76 ± 1.13	.208
MBI-GS average	2.31 ± 0.82	2.36 ± 0.81	.213

FC = frontline COVID-19, MBI-GS = Maslach Burnout Inventory-General Survey, NFC = non-frontline COVID-19.

**Table 4:** Correlation analysis among MBI-GS and UWES average values.

		MBI-GS	Dimension 1	Dimension 2	Dimension 3
UWES	Spearman correlation	−0.612	−0.417	−0.510	−0.513
	*P*	<.001	<.001	<.001	<.001
Dimension 1	Spearman correlation	−0.585	−0.451	−0.478	−0.457
	*P*	<.001	<.001	<.001	<.001
Dimension 2	Spearman correlation	−0.624	−0.422	−0.552	−0.487
	*P*	<.001	<.001	<.001	<.001
Dimension 3	Spearman correlation	−0.493	−0.292	−0.397	−0.482
	*P*	<.001	<.001	<.001	<.001

MBI-GS = Maslach Burnout Inventory-General Survey, UWES = Utrecht Work Engagement Scale.

### 3.4. Analysis of work engagement and burnout in terms of different departments

The analysis of nurse work engagement across different departments was shown in Table 5. The results of the analysis of variance indicated no significant differences among nurses from different departments in terms of UWES average, dedication dimension score, and absorption dimension score. However, a significant difference was observed in the vigor dimension score (*P* < .05). Furthermore, the analysis of nurse burnout across different departments was presented in Table 6. The results of the analysis of variance indicated significant differences among nurses from different departments in terms of emotional exhaustion, depersonalization, and personal accomplishment dimensions (*P* < .01). Additionally, we conducted a differential analysis of nurse work engagement and burnout across other demographic variables (age, gender, marital status, and educational) and the results were presented in Tables S2–S5, Supplemental Digital Content, http://links.lww.com/MD/N393, http://links.lww.com/MD/N394, http://links.lww.com/MD/N395, http://links.lww.com/MD/N396.

**Table 5:** Analysis of work engagement in terms of different departments.

Variables	Internal medicine	Surgical	Obstetrics and gynecology	Pediatrics	Emergency medicine	Levene’s variance chi-square test		ANOVA	
						Statistic	*P*	*F*	*P*
UWES average Dimension 1 Dimension 2 Dimension 3	3.24 ± 1.02 3.14 ± 1.04* 3.35 ± 1.14 3.27 ± 1.05	3.31 ± 0.99 3.23 ± 1.01† 3.45 ± 1.07 3.26 ± 1.07	3.33 ± 0.98 3.19 ± 1.01* 3.55 ± 1.02 3.30 ± 1.07	3.39 ± 0.79 3.30 ± 0.84* 3.49 ± 0.81 3.38 ± 0.92	3.36 ± 0.88 3.18 ± 0.97 3.57 ± 1.01 3.38 ± 0.96	2.204 1.501 2.035 2.059	.019 .142 .032 .030	1.442 1.969 1.535 1.196	.165 .039 .130 .293
Variables	ICU	Operating room	Psychiatry	Oncology	Other	Levene’s variance chi-square test		ANOVA	
						Statistic	*P*	*F*	*P*
UWES average Dimension 1 Dimension 2 Dimension 3	3.09 ± 0.74 2.90 ± 0.77 3.30 ± 0.90 3.10 ± 0.81	3.21 ± 0.99 3.21 ± 0.99* 3.28 ± 1.20 3.16 ± 0.99	3.37 ± 0.73 3.26 ± 0.87* 3.53 ± 0.83 3.34 ± 0.74	3.34 ± 0.96 3.24 ± 0.96 3.65 ± 1.03 3.16 ± 1.04	3.42 ± 1.05 3.33 ± 1.07† 3.55 ± 1.12 3.41 ± 1.10	2.204 1.501 2.035 2.059	.019 .142 .032 .030	1.442 1.969 1.535 1.196	.165 .039 .130 .293

UWES = Utrecht Work Engagement Scale.

* Comparison with ICU nurses, *P* < .05.

† Comparison with ICU nurses, *P* < .01.

**Table 6:** Analysis of burnout in terms of different departments.

Variables	Internal medicine	Surgical	Obstetrics and gynecology	Pediatrics	Emergency medicine	Levene’s variance chi-square test		ANOVA	
						Statistic	*P*	*F*	*P*
MBI-GS Dimension 1 Dimension 2 Dimension 3	2.48 ± 1.17 1.93 ± 1.17 2.86 ± 1.12	2.28 ± 1.05 1.82 ± 1.16 2.80 ± 1.12	2.12 ± 0.93* 1.51 ± 1.04† 2.63 ± 1.07	1.84 ± 0.89† 1.50 ± 0.77† 3.02 ± 1.01	2.22 ± 0.99 1.56 ± 1.10† 2.69 ± 0.93	1.972 1.324 4.427	.039 .219 <.001	5.282 5.213 2.242	<.001 <.001 .017
Variables	ICU	Operating room	Psychiatry	Oncology	Other	Levene’s variance chi-square test		ANOVA	
						Statistic	*P*	*F*	*P*
MBI-GS Dimension 1 Dimension 2 Dimension 3	2.49 ± 1.06 1.95 ± 1.12 2.95 ± 1.01	2.25 ± 0.99 1.93 ± 1.18 2.75 ± 1.05	2.26 ± 0.95 1.71 ± 0.82 2.95 ± 0.87	1.78 ± 1.16 1.24 ± 1.14† 2.81 ± 1.08	2.09 ± 1.16† 1.53 ± 1.09† 2.59 ± 1.31	1.972 1.324 4.427	.039 .219 <.001	5.282 5.213 2.242	<.001 <.001 .017

MBI-GS = Maslach Burnout Inventory-General Survey.

* *P* < .05 vs internal medicine nurses.

† *P* < .01 vs internal medicine nurses.

### 3.5. Logistic regression for risk factors of nurse burnout

Finally, we divided all nurses into burnout (MBI-GS average value > 2.5) and non-burnout (MBI-GS average value < 2.5) groups based on the average MBI-GS values^[8]^ and used the entry method for logistic regression to analyze the risk factors of nurse burnout. For logistic regression, we used three models using the entry method, including model 1 (age, sex, marital status, working age, daily overtime, weekly rest time, night shifts, fight against COVID-19, and smoking or drinking), model 2 (GHQ-12 score and average values of each dimension of UWES). The results showed that daily overtime (95%CI 2.371–367.446, *P* = .009), fight against COVID-19 (95%CI 1.012–1.590, *P* = .039), GHQ-12 score (95%CI 0.501–0.812, *P* < .001) and dimension 2 of UWES (95%CI 0.321–0.525, *P* < .001) were the risk factors for nurse burnout (Table 7).

**Table 7:** Logistic regression for risk factors of nurse burnout.

Variables	Wald	Odds ratio	95%CI	*P*
Model 1				
Age	1.178	0.984	0.955–1.013	.278
Sex	2.865	1.657	0.923–2.973	.091
Marital status	0.031	0.977	0.756–1.263	.859
Working age	1.214	0.905	0.757–1.081	.271
Daily Overtime	32.124	1.486	1.296–1.705	<.001
Weekly rest time	1.499	0.832	0.620–1.117	.221
Night shifts	3.705	1.233	0.996–1.527	.054
Fight against COVID-19	4.260	1.269	1.012–1.590	.039
Smoking or drinking	2.407	0.570	0.281–1.159	.121
Education	0.004	1.007	0.794–1.279	.952
Model 2				
GHQ-12	37.00	0.638	0.501–0.812	.021
Dimension 1 of UWES	2.547	0.809	0.623–1.050	.110
Dimension 2 of UWES	50.667	0.410	0.321–0.525	<.001
Dimension 3 of UWES	1.140	1.128	0.905–1.405	.286

GHQ-12 = General Health Questionnaire-12, UWES = Utrecht Work Engagement Scale.

## 4. Discussion

The consequences of nurse burnout are significant and far-reaching. It can lead to high turnover rates, decreased job performance, and compromised patient safety. It can also affect nurses’ mental and physical health, leading to increased absenteeism, substance abuse, and depression.^[23,24]^ Therefore, it is urgent to understand the risk factors for nurse burnout. In this investigation study, we found that daily overtime, fight against COVID-19, GHQ-12 score and dimension 2 of UWES were the risk factors of nurse burnout.

Many previous studies have focused on the risk factors of nurse burnout. Galanis et al found that burnout levels were highest among men, individuated who were single or divorced, and those without children and nurses. Additionally, these relationships may be exacerbated by moderating variables such as age, qualifications, and job satisfaction.^[19]^ A pre-COVID-19 meta-analysis confirmed a significant relationship between dimensions of occupational burnout, such as emotional exhaustion, depression, and personality factors. Sociodemographic factors (such as youth, marital status, and less experience in the intensive care unit) and working conditions (such as high workload and long working hours) were found to potentially influence the risk of developing burnout syndrome.^[25]^ However, in our study, we found no association between factors such as age and marital status and nurse burnout, which may be attributed to differences in race and geographic location of the study population. In the context of the COVID-19 pandemic, the increased workload, exposure to high-risk situations, and emotional strain associated with caring for COVID-19 patients may overshadow the influence of traditional sociodemographic factors on nurse burnout. The unprecedented demands and stressors brought about by the pandemic may have a more substantial impact on the development of burnout in nurses.^[26,27]^

In our study, we found that daily overtime hours were a risk factor for nurse burnout. Before the COVID-19 pandemic, Luther et al’s study also yielded similar results, as they found that clinical physicians who worked overtime reported significantly higher levels of job burnout and work-life conflict, as well as significantly lower levels of job satisfaction and quality of care.^[28]^ The studies conducted by Wheatley^[29]^ and Naylor et al^[30]^ also confirmed this conclusion, as they both found a link between overtime and nurse burnout. In addition, Uchmanowicz et al investigated 594 nurses and found a positive correlation between the Basel Extent of Rationing of Nursing Care-R (BERNCA-R) scores and the Maslach Burnout Inventory (MBI) scores, while they were negatively correlated with the Job Satisfaction Scale (JSS) scores. Occupational burnout was found to decrease job satisfaction among healthcare professionals and lead to adverse consequences of rationing care.^[31]^ Similarly, in our study, we also observed a negative correlation between the average scores of MBI-GS dimensions and the overall average score of UWES, with the dedication dimension of UWES being identified as a risk factor for nurse burnout.

We also analyzed the relationship between first against COVID-19 and nurse burnout. COVID-19 is considered the cause of a dangerous disease that affects people’s lives and, in many cases, threatens the lives of those infected.^[32]^ Whether in everyday life or in times of disaster, nurses are on the front lines, responsible for providing holistic care for a variety of patients.^[18,33]^ This also makes nurses a high-risk group for COVID-19 infection. A meta-analysis of data as of May 8, 2020 found that 25.3% of healthcare workers who died from COVID-19 were nurses. Therefore, it is necessary to analyze whether FC response is causing nurse burnout.^[34]^ In our study, we found that the dimension 1 (emotional exhaustion) average value and dimension 2 (depersonalization) average value of MBI-GS in NFC group were remarkably declined than that in FC group and fight against COVID-19 was the risk factors of nurse burnout.

Nurse burnout is a syndrome characterized by three aspects: emotional exhaustion, depersonalization, and reduced personal accomplishment.^[35]^ The COVID-19 pandemic has led to an increase in emotional exhaustion among nurses due to fear of infection, long working hours, and high patient loads. Depersonalization is also an issue as nurses may become insensitive to patients due to the need to provide care in a highly controlled environment. Reduced personal accomplishment is also a risk as nurses may feel that their efforts have not made a difference in the face of the overwhelming nature of the pandemic. These reasons may lead to risk factors for nurse burnout in the context of FC response. Some other studies have yielded similar results to ours. Murat et al found more burnout among nurses who tested positive for COVID-19 and those who did not wish to volunteer during the pandemic.^[36]^ Another study in Wuhan showed that 288 (14.3%), 217 (10.7%), and 1837 (91.2%) nurses reported moderate to high levels of anxiety, depression, and fear, respectively, which can directly lead to nurse burnout.^[37]^ Factors such as personal protective equipment shortages, fear of contracting the virus, long shifts, and witnessing significant morbidity and mortality among patients may contribute to heightened levels of emotional exhaustion and depersonalization. The intense and prolonged nature of the pandemic, coupled with the uncertainty and constant changes in guidelines and protocols, can exacerbate feelings of burnout among nurses.^[38,39]^ It is essential to recognize the unique challenges posed by the pandemic and develop targeted interventions and support systems to address nurse burnout in this context. Adequate staffing, access to mental health resources, regular communication, and supportive leadership are crucial in mitigating the impacts of COVID-19 on nurse burnout. Therefore, preventive and promotive interventions for psychological well-being should be planned and implemented to improve the situation of nurse burnout during the pandemic, and to prepare nurses who may work during future pandemics.

This study also has several limitations. Firstly, it was conducted in Wuhan, China, which was severely affected by COVID-19. Therefore, the generalizability of the study findings to regions with different healthcare systems or pandemic experiences may be limited. Secondly, the study relied on self-report data, which may introduce response biases or inaccuracies. Thirdly, the factors analyzed in this study may not cover all possible influences. For instance, economic pressures or health issues outside of work could also impact nurse’s burnout.

## 5. Conclusion

In summary, this study showed that the dimension 1 (emotional exhaustion) average value and dimension 2 (depersonalization) average value of MBI-GS in NFC group were remarkably declined than that in FC group. In the future, interventions at the national and organizational levels will be necessary to provide sufficient social support and ensure frontline work willingness to improve the situation of nurse burnout.

## Author contributions

**Data curation:** Xia Wang.**Formal analysis:** Ying Han.

**Writing – original draft:** Kunyu Wang, Chunfen Ye, Lifen Pan.

**Writing – review & editing:** Changcai Zhu, Ying Han.

## Supplementary Material
