# Supplementary material for: The risk factors for burnout among nurses: An investigation study
Source: Medicine (Baltimore). 2024 Aug 23;103(34):e39320. doi: 10.1097/MD.0000000000039320 (PMC11346864; doi:10.1097/MD.0000000000039320)
Supplement: Supplementary file 1 [file medi-103-e39320-s001.docx]

**Supporting information of the risk factors for burnout among nurses: an investigation study**

**Supplementary table 1. General demographic information**

| Variables | Category | Total (n=1712) | |
| --- | --- | --- | --- |
|  |  | Numbers (n) | Proportions (%) |
| Hospital  Department  Age  Sex  Marital status  Education  Length of service  Employment form  Title  Position  Daily Overtime  Weekly rest time  Night shifts per month | Tianyou Hospital affiliated to Wuhan University of Science and Technology  Wuhan Taikang Tongji Hospital  Hankou Hospital of Wuhan  Liyuan Hospital affiliated to Tongji Medical College  Hubei Provincial Hospital of Integrated Traditional Chinese and Western Medicine  Third People's Hospital of Hubei Province  Internal Medicine  Surgical  Obstetrics and Gynecology  Pediatrics  Emergency Medicine  ICU  Operating Room  Psychiatry  Oncology  *other  ≤25 y  26-30 y  31-35 y  36-40 y  41-45 y  ≥46  Male  Female  Married  Unmarried  Divorced  Widowed  Junior college or below  Bachelor or above  1-3 y  4-5 y  6-10 y  11-20 y  >20 y  Formal preparation  Contract based system  Other  Nurse  Nurse Practitioner  Nurse Practitioner in Charge  Associate Nurse Practitioner and above  No position  Nursing team leader/specialist nurse/teaching nurse  Nurse head  Chief Nurse/Director of Nursing Department  0h  0.5-1 h  1.5-2 h  >2 h  <2 d/week  2 d/week  >2 d/week  0 time/month  1-5 times/month  6-10 times/month  >10 times/month | 327  231  253  251  351  299  520  379  93  58  83  108  71  26  67  307  411  540  360  230  80  91  55  1657  714  969  27  2  410  1302  331  254  504  451  172  247  1425  40  498  738  430  46  1287  272  137  16  371  1062  176  103  180  1503  29  571  384  519  238 | 19.10  13.49  14.78  14.66  20.50  17.47  30.39  22.14  05.43  03.39  04.85  06.31  04.15  01.52  03.91  17.93  24.00  31.54  21.03  13.44  04.67  05.32  03.21  96.79  41.70  56.60  01.58  00.12  23.95  76.05  19.33  14.84  29.44  26.34  10.05  14.43  83.23  02.34  29.08  43.11  25.12  02.69  75.18  15.89  08.00  00.93  21.67  62.03  10.28  06.02  10.51  87.80  01.69  33.35  22.43  30.32  13.90 |

* Other departments include outpatient clinics, nursing departments, hemodialysis centers, Chinese medicine departments, rehabilitation departments, sterilization supply centers, medical imaging departments, health management centers and other nursing units
