# Supplementary material for: The risk factors for burnout among nurses: An investigation study
Source: Medicine (Baltimore). 2024 Aug 23;103(34):e39320. doi: 10.1097/MD.0000000000039320 (PMC11346864; doi:10.1097/MD.0000000000039320)
Supplement: Supplementary file 2 [file medi-103-e39320-s002.docx]

**Supporting information of the risk factors for burnout among nurses: an investigation study**

**Supplementary table 2. Analysis of nurses' work engagement and burnout in terms of gender**

| Variables | Male | Female | *P* |
| --- | --- | --- | --- |
| UWES average  Dimension 1  Dimension 2  Dimension 3  MBI-GS  Dimension 1  Dimension 2  Dimension 3 | 3.42±1.01  3.34±1.04  3.58±1.20  3.37±1.04  2.15±0.99  1.65±1.18  2.64±1.19 | 3.29±0.97  3.19±1.00  3.43±1.07  3.29±1.03  2.29±1.11  1.76±1.13  2.79±1.13 | 0.353  0.282  0.330  0.545  0.348  0.452  0.349 |

MBI-GS, Maslach Burnout Inventory-General Survey; UWES, Utrecht Work Engagement Scale.
