# Supplementary material for: The risk factors for burnout among nurses: An investigation study
Source: Medicine (Baltimore). 2024 Aug 23;103(34):e39320. doi: 10.1097/MD.0000000000039320 (PMC11346864; doi:10.1097/MD.0000000000039320)
Supplement: Supplementary file 3 [file medi-103-e39320-s003.docx]

**Supporting information of the risk factors for burnout among nurses: an investigation study**

**Supplementary table 3. Analysis of nurses' work engagement and burnout in terms of age**

| Variables | ≤25 y | 26-30 y | 31-35 y | 36-40 y | 41-45 y | ≥46 y | Levene's variance chi-square test | | ANOVA | |
| --- | --- | --- | --- | --- | --- | --- | --- | --- | --- | --- |
|  |  |  |  |  |  |  | statistic | P | F | P |
| UWES average  Dimension 1  Dimension 2  Dimension 3  MBI-GS  Dimension 1  Dimension 2  Dimension 3 | 3.27±1.00  3.15±1.05  3.44±1.08  3.25±1.03  2.28±1.09  1.78±1.11  2.98±1.06 | 3.17±0.98  3.07±0.99  3.32±1.07  3.15±1.03  2.38±1.10  1.84±1.12  2.93±1.05 | 3.34±0.93  3.22±0.95  3.47±1.05  3.35±0.97  2.28±1.11  1.78±1.21  2.72±1.12 | 3.39±0.97  3.31±1.00  3.48±1.05  3.38±1.06  2.26±1.11  1.66±1.08  2.47±1.21 | 3.68±0.93  3.60±0.89  3.81±1.08  3.64±1.07  2.18±1.20  1.53±1.10  2.31±1.18 | 3.52±0.98  3.47±0.99  3.62±1.15  3.48±1.02  1.94±0.98  1.52±1.05  2.51±1.32 | 0.137  0.470  0.193  0.453  0.646  0.547  7.311 | 0.984  0.799  0.965  0.811  0.665  0.741  0.000 | 1.333  1.504  1.168  1.310  0.937  0.192  0.520 | 0.088  0.027*  0.226  0.101  0.456  0.966  0.761 |

**P*<0.05；***P*<0.01. MBI-GS, Maslach Burnout Inventory-General Survey; UWES, Utrecht Work Engagement Scale.
