# Supplementary material for: The risk factors for burnout among nurses: An investigation study
Source: Medicine (Baltimore). 2024 Aug 23;103(34):e39320. doi: 10.1097/MD.0000000000039320 (PMC11346864; doi:10.1097/MD.0000000000039320)
Supplement: Supplementary file 4 [file medi-103-e39320-s004.docx]

**Supporting information of the risk factors for burnout among nurses: an investigation study**

**Supplementary table 4. Analysis of nurses' work engagement and burnout in terms of marital status**

| Variables | Unmarried | Married | Divorced/widowed | Levene's variance chi-square test | | ANOVA | |
| --- | --- | --- | --- | --- | --- | --- | --- |
|  |  |  |  | statistic | P | F | P |
| UWES average  Dimension 1  Dimension 2  Dimension 3  MBI-GS  Dimension 1  Dimension 2  Dimension 3 | 3.20±0.98  3.07±1.02  3.36±1.07  3.18±1.03  2.31±1.10  1.79±1.10  2.96±1.06 | 3.37±0.97^##^  3.28±0.99^##^  3.49±1.07^#^  3.36±1.02^##^  2.27±1.10  1.74±1.15  2.67±1.17^##^ | 3.46±1.01  3.33±1.00  3.60±1.20  3.48±1.05  2.06±1.04  1.56±1.04  2.54±1.02 | 0.098  0.037  0.487  0.332  0.129  0.232  8.739 | 0.907  0.964  0.615  0.717  0.879  0.793  0.000 | 7.148  9.193  3.226  7.087  0.886  0.929  14.679 | 0.001**  0.000**  0.040*  0.001**  0.412  0.395  0.000** |

*P<0.05; **P<0.01; # compared with unmarried group of nurses, P<0.05; ## compared with unmarried group of nurses, P<0.01. MBI-GS, Maslach Burnout Inventory-General Survey; UWES, Utrecht Work Engagement Scale.
