# Supplementary material for: The risk factors for burnout among nurses: An investigation study
Source: Medicine (Baltimore). 2024 Aug 23;103(34):e39320. doi: 10.1097/MD.0000000000039320 (PMC11346864; doi:10.1097/MD.0000000000039320)
Supplement: Supplementary file 5 [file medi-103-e39320-s005.docx]

**Supporting information of the risk factors for burnout among nurses: an investigation study**

**Supplementary table 5. Analysis of nurses' work engagement and burnout in terms of education**

| Variables | Junior college or below | Bachelor or above | *P* |
| --- | --- | --- | --- |
| UWES average  Dimension 1  Dimension 2  Dimension 3  MBI-GS  Dimension 1  Dimension 2  Dimension 3 | 3.30±0.98  3.19±1.01  3.49±1.10  3.26±1.03  2.27±1.11  1.72±1.08  2.84±1.12 | 3.30±0.97  3.20±1.00  3.42±1.07  3.30±1.03  2.29±1.10  1.77±1.15  2.77±1.13 | 0.914  0.542  0.966  0.782  0.672  0.649  0.138 |

MBI-GS, Maslach Burnout Inventory-General Survey; UWES, Utrecht Work Engagement Scale.
